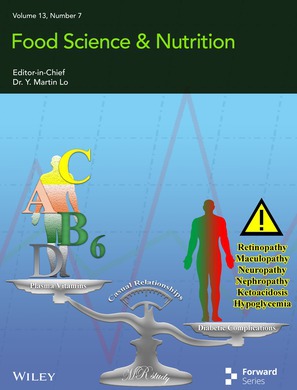# Cover Image

**DOI:** 10.1002/fsn3.70618

**Published:** 2025-08-06

**Authors:** Sijia Cai, Weitao Man, Wenqing Liu, Bowu Li, Zhongchen He, Guman Duan

## Abstract

The cover image is based on the article *Causal Relationship Between Various Vitamins and Different Diabetic Complications: A Mendelian Randomization Study* by Guman Duan et al., https://doi.org/10.1002/fsn3.70536.